# Supplementary material for: Unicolored phosphor-sensitized fluorescence for efficient and stable blue OLEDs
Source: Nat Commun. 2018 Nov 26;9:4990. doi: 10.1038/s41467-018-07432-2 (PMC6255836; doi:10.1038/s41467-018-07432-2)
Supplement: Supplementary file 1 — Supplementary Information [file 41467_2018_7432_MOESM1_ESM.docx]

Supplementary information

**Unicolored phosphor-sensitized fluorescence for efficient and stable blue OLEDs**

Paul Heimel,^1,2,3^ Anirban Mondal,^4^ Falk May,^3,§^ Wolfgang Kowalsky,^1,2^ Christian Lennartz,^1,3,†^ Denis Andrienko,^4,#^ and Robert Lovrincic^1,2,#^

^1^InnovationLab, 69115 Heidelberg, Germany
^2^Institute for High Frequency Technology, TU Braunschweig, 38106 Braunschweig, Germany
^3^BASF SE, 67056 Ludwigshafen, Germany
^4^Max Planck Institute for Polymer Research, 55128 Mainz, Germany
^†^Present address: trinamiX GmbH, 67063 Ludwigshafen, Germany
^§^Present address: Merck KGaA, 64293, Darmstadt, Germany

^#^to whom correspondence should be addressed:
denis.andrienko@mpip-mainz.mpg.de, r.lovrincic@tu-braunschweig.de

# Estimation of the Förster radius

The Förster radius $R_{\mathrm{FRET}}$ was estimated from the overlap $J\left( \lambda\right)$ of the extinction spectrum of the acceptor (A) and photoluminescence spectrum of the donor (D)^25^

$R_{\mathrm{FRET}}^{6}=\frac{9000\ln10\kappa^{2}\Phi_{D}}{128\pi^{5}N_{A}n^{4}}J\left( \lambda\right),$ (1)

where $\Phi_{D}$ is the donor’s quantum yield, $\kappa$ the orientation factor, and $n$ the refractive index of the medium. We used $\Phi_{D}=1$, $\kappa$^2^ = 0.476 ^52^, and $n = 1.7$. The extinction spectra of the acceptor in toluene, which are shown for different concentrations in **Supplementary Figure 1** together with the PL spectrum of the donor, yield $J\left( \lambda\right)=3.8\times{10}^{13}$ M^-1^ cm^-1^ nm^4^, $R_{\mathrm{FRET}}=2.4 \mathrm{nm}$, as mentioned in the main text.


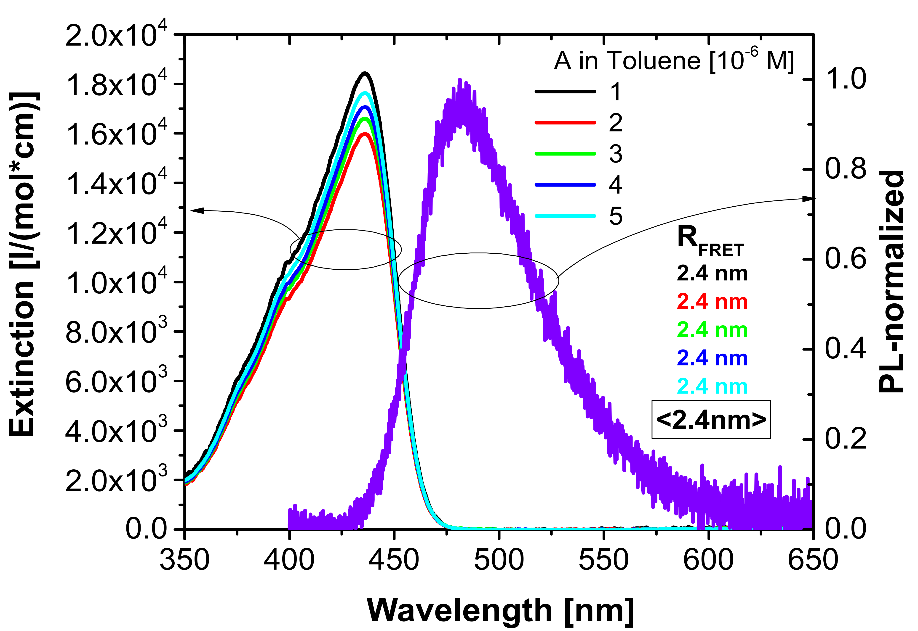


**Supplementary Figure 1** Extinction spectra of acceptor and donor photoluminescence spectrum. Acceptor was dissolved in toluene at different concentrations. The donor photoluminescence spectrum is intensity- normalized. The overlap of these spectra results in an average Förster radius of 2.4 nm.

# Fluorescence and radiative decay times

To estimate the average (intensity-weighted) fluorescence decay time, we performed the multi-exponential fits of the TRPL spectra of the mixed layers with total of 4 exponentials,

$I\left( t \right)=\sum_{i=1}^{4} A_{i}e^{-\frac{t}{\tau_{i}}}$ , (2)

where $\sum_{i=1}^{4} A_{i}=1$. The fits are shown in **Figure 3** of the main text, and the fit parameters are summarized in the table below.

| A (%) | $A_{1}$ | $A_{2}$ | $A_{3}$ | $A_{4}$ | $\tau_{1}$[µs] | $\tau_{2} [\mu s]$ | $\tau_{3}$[µs] | $\tau_{4}$[µs] |
| --- | --- | --- | --- | --- | --- | --- | --- | --- |
| 0.5 | 0.209 | 0.181 | 0.230 | 0.399 | 1.098 | 0.377 | 0.084 | 0.015 |
| 1.0 | 0.095 | 0.181 | 0.276 | 0.451 | 0.801 | 0.296 | 0.070 | 0.013 |
| 1.5 | 0.047 | 0.161 | 0.290 | 0.506 | 0.621 | 0.233 | 0.063 | 0.012 |

**Supplementary Table 1** TRPL spectra fit parameters.

The average (intensity-weighted) fluorescence decay time $\tau_{fl}$ was then calculated as $\tau_{fl}=\frac{\sum_{i=1}^{4} {A_{i}\tau_{i}}^{2}}{\sum_{j=1}^{4} A_{j}\tau_{j}}$. The corresponding values are summarized in the table below.

| A (%) | 0.5 | 1.0 | 1.5 |
| --- | --- | --- | --- |
| $\tau_{fl}$ [µs] | 0.87 | 0.51 | 0.31 |

**Supplementary Table 2** Average fluorescence decay times.

The radiative decay times were then obtained by weighting the fluorescence decay times with the corresponding quantum yields, $\tau_{r}=\tau_{fl}/\Phi_{PL}$.^53^ Indeed, the photoluminescence quantum yield $\Phi_{PL}$ in a system with quenching species can be determined as

$\Phi_{PL} = \frac{k_{r}}{k_{r} + k_{nr} + k_{DQ}}$ (3)
where $k_{r}$ is the radiative decay rate, $k_{nr}$ is the non-radiative decay rate, and $k_{DQ}$ is the Dexter quenching rate. The fluorescent decay time is given by

$\tau_{fl}=\frac{1}{k_{r} + k_{nr} + k_{DQ}}$ (4)
where $\tau_{fl}$ can be extracted from the TRPL-spectra. The ratio of the photoluminescence quantum yield and the fluorescence decay time gives then the radiative decay rate, $\frac{\Phi_{PL}}{\tau_{fl}}=k_{r}$. The calculated radiative decay times are summarized in Table 1 of the main text.

# Estimation of the Förster rate

The FRET-rates $k_{FRET}$ of the mixed layers were estimated from the radiative decay rates according to the following approximation:

$k_{r}=\tau_{r}^{-1}\approx k_{ph}+k_{FRET}$ (5)
where $k_{ph}$ is the radiative decay rate of the donor in absence of the acceptor.

The transfer efficiencies, $E_{FRET}$, giving the proportion of excitons decaying radiatively due to FRET to the acceptor, are given by:

$E_{FRET}=\frac{k_{FRET}}{k_{ph}+k_{FRET}}$ (6)

The calculated values are summarized in the table below.

| acceptor (%) | 0.5 | 1.0 | 1.5 |
| --- | --- | --- | --- |
| $k_{FRET}$ [10^6^ s^-1^] | 0.32 | 0.67 | 1.42 |
| $E_{FRET}$ | 0.34 | 0.52 | 0.69 |

**Supplementary Table 3** Estimated Förster resonant transfer rates and transfer efficiencies.

# PL response of the acceptor

The excitation spectra of the acceptor, **Supplementary Figure 2**, shows that the lowest PL-response of the acceptor is at 375 nm. This wavelength was used to excite mixed bilayers to ensure predominant excitation of donor molecules, as mentioned in the main text. At 355 nm wavelength, PLQY is of the order of 95 %.


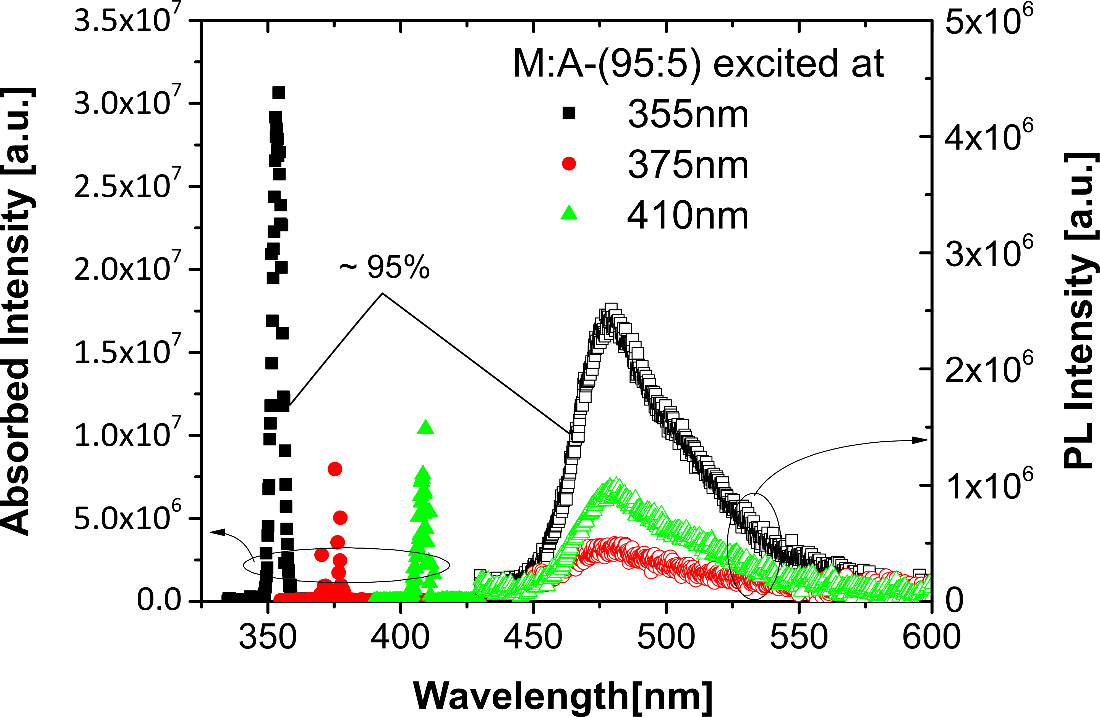


**Supplementary Figure 2** Excitation spectra of the matrix:acceptor layer (5 % doping concentration) on glass. The sample was excited at 355 nm, 375 nm and 410 nm respectively in order to find the lowest acceptor response.

# PLQY measurements of mixed layers

PL-spectra of mixed UPSF-samples, which corresponds to the time-resolved PL measurements, **Figure 3**(b) of the main text, are shown in **Supplementary Figure 3.** The sample with donor only has quantum yield of $\sim100\%$. Similarly, an emitter layer with acceptor only has PLQY of 95%. For mixed layers, the PLQY gradually decreases with the increase of the acceptor concentration, which we interpret as an increase of the Dexter transfer from the excited triplets of the donor to the triplets of the fluorescent acceptor, where they are subsequently trapped and quenched.


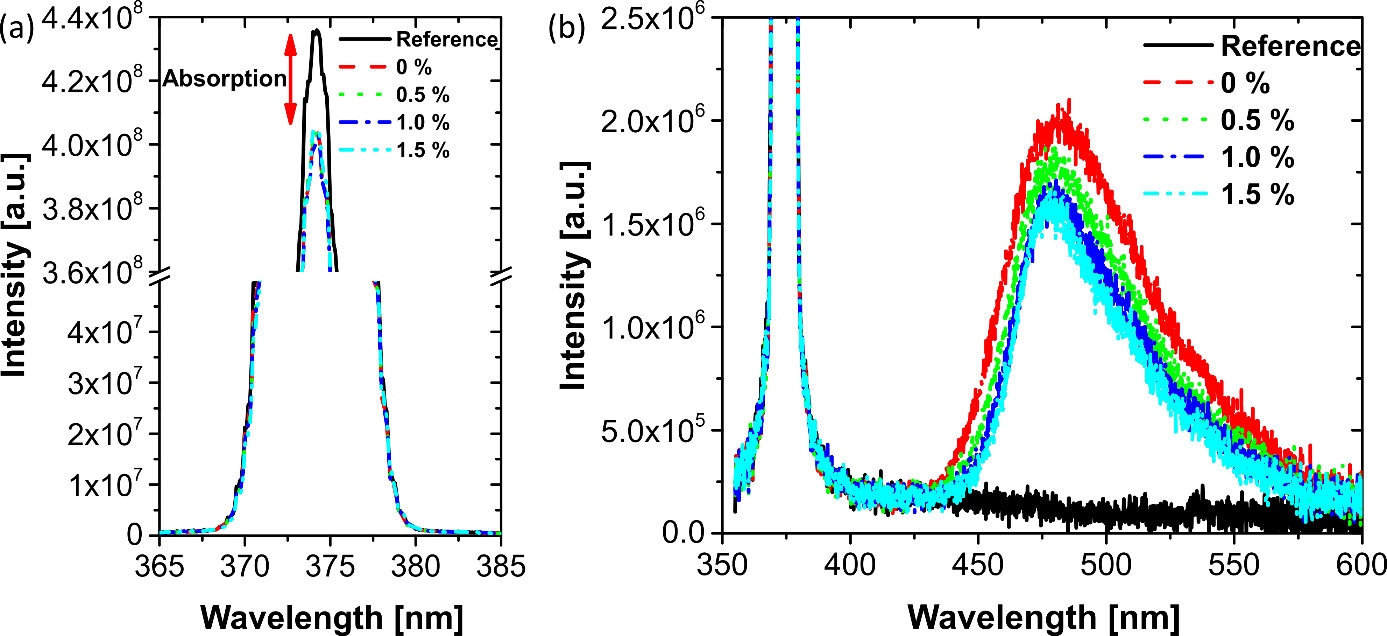


**Supplementary Figure 3** Steady-state photoluminescence spectra of matrix:donor:acceptor layers. Measurements are on a glass substrate, excitation at 375 nm. The acceptor concentration was gradually increased from 0 % to 1.5 %. The excitation signal is shown in (a). The red arrow indicates the difference (absorption) of the detected excitation signal between the blank substrate (Reference) and each layer on the substrate. The photoluminescence response is shown in (b).

# Simulations

To convert volume % into number densities, we first equilibrated cubic boxes of 3000 molecules of pure materials. The corresponding box volumes, V (acceptor) = 3819 nm^3^, V (host) = 2157 nm^3^, V (donor) = 3507 nm^3^, were used to calculate the volume per molecule, 1.273 nm^3^ per acceptor, 0.719 nm^3^ per host, and 1.169 nm^3^ donor molecule. The number of acceptor (A), matrix (M), and donor (D) molecules in a mixture with the 0.5:79.5:20 volume fractions were then obtained from the ratios 1.273A : 0.719M : 1.169D = 0.5:79.5:20. This implies that the molecular ratio should be 1 : 281 : 44 for acceptor : matrix : donor, e.g. 80 acceptor + 22480 host + 3520 donor molecules, in a simulation box containing 26080 molecules.

A summary of the studied systems is provided in **Supplementary Table 4**. We simulated two different system sizes (small and 8 times larger systems). In addition to experimental volume fractions of 0.5 %, 1.0 % and 1.5 % of acceptor, four other small systems with different mole ratios were simulated to get a concentration dependence of Forster and Dexter transfer rates.

| Volume fraction as in experiment  (acceptor : host : donor) | Number of molecules | | | Volume (nm^3^) |
| --- | --- | --- | --- | --- |
|  | Acceptor | Host | Donor |  |
| a) 0.5 : 79.5 : 20 | 80 (10) | 22480 (2810) | 3520 (440) | 21906.379 (2741.414) |
| b) 1.0 : 79.0 : 20 | 160 (20) | 22400 (2800) | 3520 (440) | 22000.275 (2753.448) |
| c) 1.5 : 78.5 : 20 | 240 (30) | 22320 (2790) | 3600 (450) | 22091.675 (2761.749) |
| --- | --- (25) | --- (3000) | --- (450) | --- (2762.040) |
| --- | --- (40) | --- (3000) | --- (450) | --- (2776.289) |
| --- | --- (50) | --- (3000) | --- (450) | --- (2789.977) |
| --- | --- (60) | --- (3000) | --- (450) | --- (2800.970) |

**Supplementary Table 4** System specification used in all-atom molecular dynamics simulations of acceptor, host and donor bulk mixtures. Numbers in parentheses are for smaller system size.


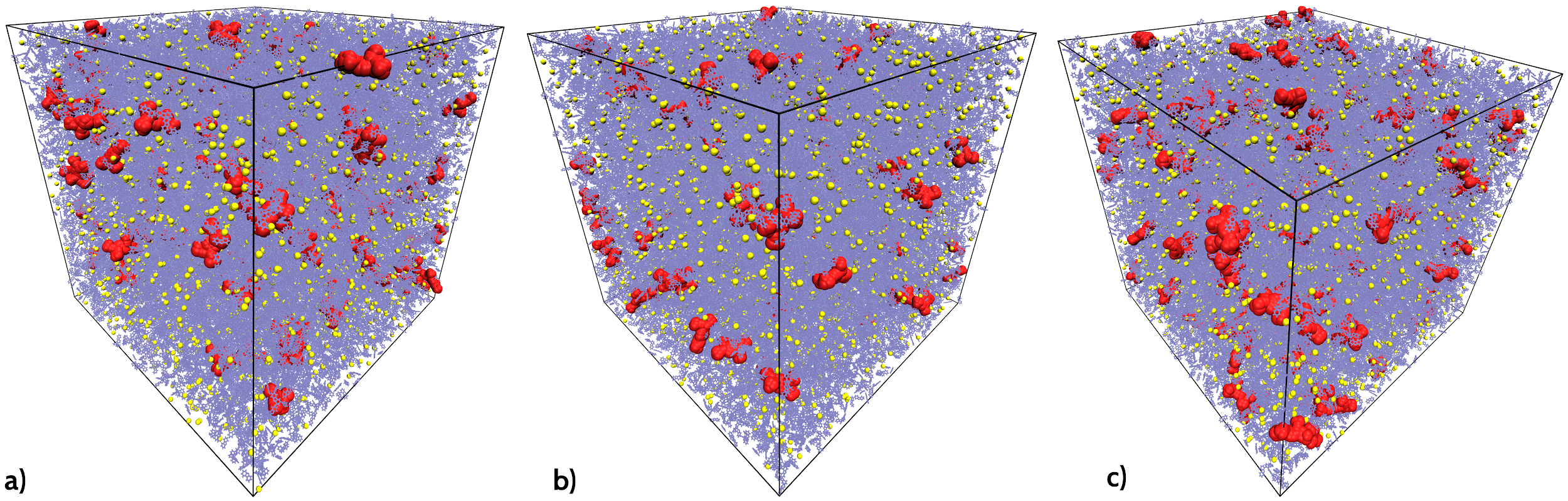


**Supplementary Figure 4** Snapshots of molecular dynamics simulations. The acceptor-host-donor bulk mixtures for three different concentrations of acceptor (0.5 volume %, 1.0 % and 1.5 %) are shown. Acceptor is shown in red, donor (only the central atom) in yellow. System sizes are described in Table 1.

Typical MD snapshots for three acceptor concentrations are shown in **Supplementary Figure 4**. One can see that both donor and acceptor are uniformly distributed through the box and randomly oriented. To extract relevant to FRET distances, we calculated the number of acceptor molecules at a center-of-mass distance $r$ from every donor molecule. The averaged over all donors values, $N_{A}(r)$ are shown in **Supplementary Figure 5**. These profiles scale as $r^{2}$ (volume of spherical shells around a donor) and superimpose on a single curve when weighted by the acceptor concentration.


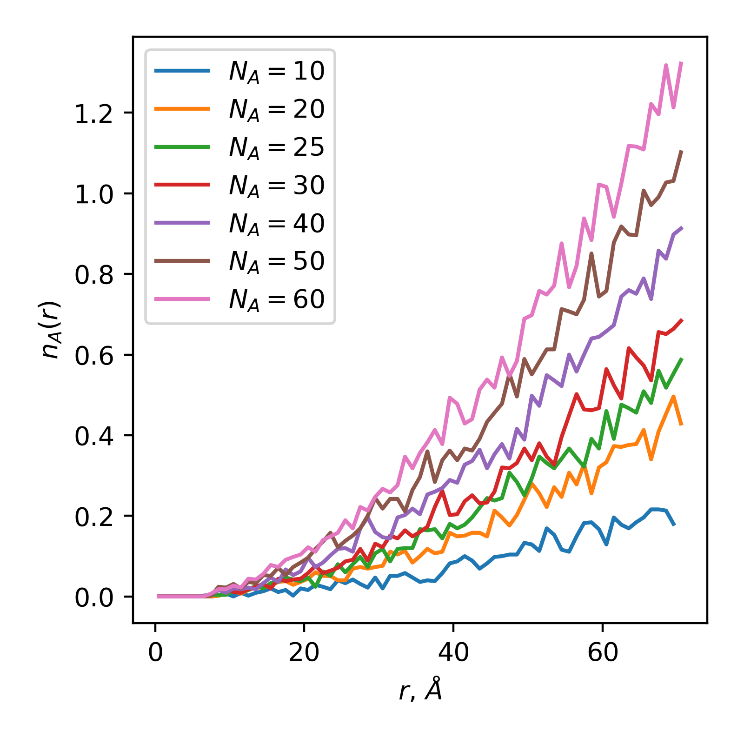


**Supplementary Figure 5** Average number of acceptor molecules at a distance *r* from a donor molecule. $N_{A}=10$ corresponds to 0.5 vol %.

For Dexter transfer, for every donor molecule we have calculated the number of acceptors molecules with the closest distance of 0.6 and 0.8 nm. These donor-acceptor pairs are likely to participate in the Dexter transfer. **Supplementary Figure 6** shows the number of donor molecules with a neighboring acceptor as a fraction of total donor molecules in the system. One can see that, for experimentally relevant concentrations, the number of quenched by Dexter transfer donors grows linearly with the acceptor concentration. For 1 volume % of acceptors ($N_{A}=20$) around 10 % of donor molecules are quenched. This is the first indication that UPSF can be significantly improved by designing donor-acceptor pairs with a reduced Dexter rate, as mentioned in the main text.


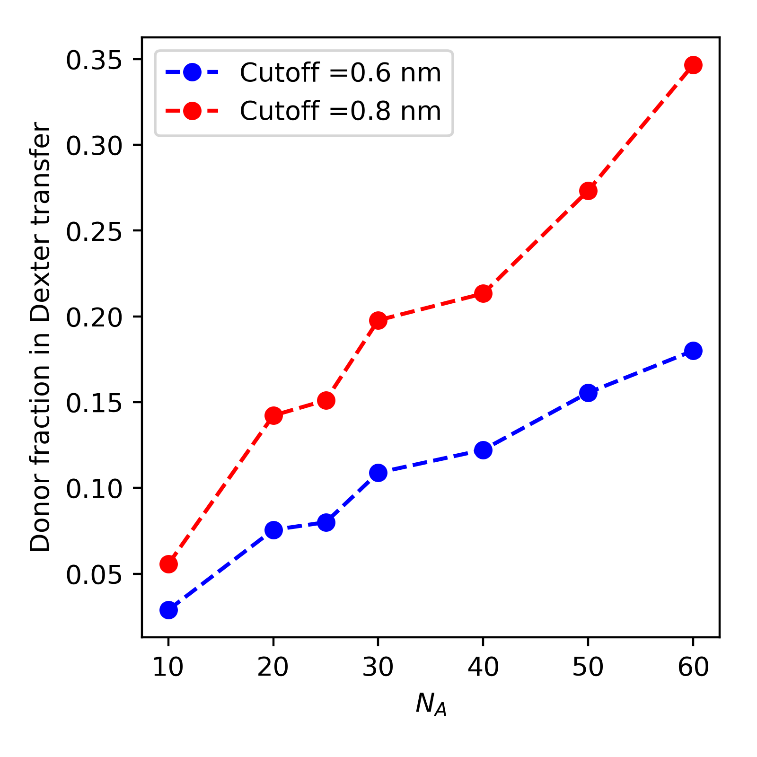


**Supplementary Figure 6** Percent of donor molecules participating in the Dexter transfer. The dependence is shown for two cutoff distances between donor-acceptor centers of mass. $N_{A}=10$ corresponds to 0.5 vol %.

Finally, we have evaluated the average Förster energy transfer rate by evaluating the sum over all acceptors, $k_{\mathrm{FRET}}=k_{\mathrm{Ph}}\sum_{\mathrm{acceptors}} \left( \frac{R_{\mathrm{FRET}}}{R_{\mathrm{DA}}} \right)^{6}$. From this sum we excluded all acceptors participating in the Dexter transfer. The $1/R^{6}$ decay of the Dexter rate is known to overestimate the FRET rate (since it relies on the dipole-dipole approximation). To correct for this, we scaled all rates by a factor of 5, which then gives a reasonable agreement with experimentally estimated rates, as shown in **Supplementary Figure 7**. One can see that we can still increase the FRET rate by increasing the concertation of the acceptor, but this increase quickly saturates at ca 3 vol %. Significantly larger boost can be obtained by reducing the Dexter rate, which might lead to up to a factor 2 increase in the average FRET rate and hence boost the lifetime of the device without compromising its efficiency.


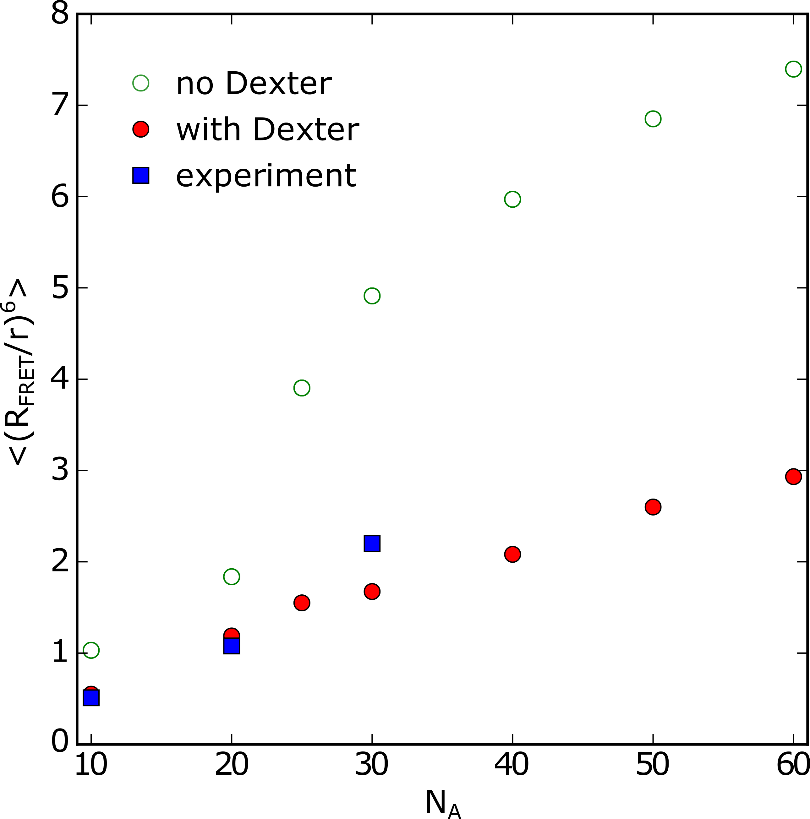


**Supplementary Figure 7** Average Förster rate with Dexter quenching taken into account and without it. Suppressing Dexter transfer would lead to at least a factor of two increase in the average Förster rate. $N_{A}=10$ corresponds to 0.5 vol %.

# OLED stack and materials

The UPSF-OLEDs fabricated are based on a state-of-the-art OLED stack (see **Supplementary Figure 8** for the OLED stack and materials used). Under operation, charge carriers are injected into the organic semiconducting materials by the aluminum cathode (electrons) and the transparent ITO anode (holes). For improved injection and charge transport, doped transport materials for both electrons (ETL) and holes (HTL) and an additional injection layer on the electron side (EIL) were used. To prevent charge carrier leakage from the emission layer (EML), HTM and matrix were used as electron blocking (EBL) and hole blocking (HBL) layers respectively. Thereby, exciton formation is restricted to the EML. Furthermore, both HTM and matrix possess higher triplet energies to avoid energy transfer to those materials (exciton quenching). The emitter molecules are embedded in a Matrix M, to avoid self-quenching and reduce polaron-stress on the emitter. To reduce the number of holes conducted by the emitter molecules and restrict exciton formation to the donor molecules, we used an additional hole transport material in the EML. As a result, the amount of the matrix material was slightly reduced, with an overall doping ratio in the EML of 70-$c_{A}$:20:10:$c_{A}$ for M:D:HTM:A, where $c_{A}$ is the doping concentration of the acceptor. This can be explained by the higher doping concentration of the HTM and its large electric dipole moment of 8 Debye,^54^ which supports trapping of the holes on HTM.^55^

For the synthesis of the not purchasable compounds, we refer to the following literature: D^30^, A^31^ (the difference is only in the substitution of the side groups, 1,8 instead of 1,6), M^32^, ETM^56^. Electron Affinities and Ionization potentials were obtained by density functional theory. Neutral and charged molecules were geometry-optimized in the gas phase using the generalized gradient approximation functional of Becke^57^ and Perdew (BP86)^58^ in combination with polarized double-zeta valence (def2-SVP) basis sets as implemented in the TURBOMOLE package.^59^ Energy differences between neutral and charged states were then evaluated on the optimized structures with the same functional but a polarized triple-zeta valence (def2-TZVP) basis within the conductor-like screening model (COSMO)^60^ which treats the environment as a dielectric continuum of relative permittivity $\epsilon_{r}=4.5$.


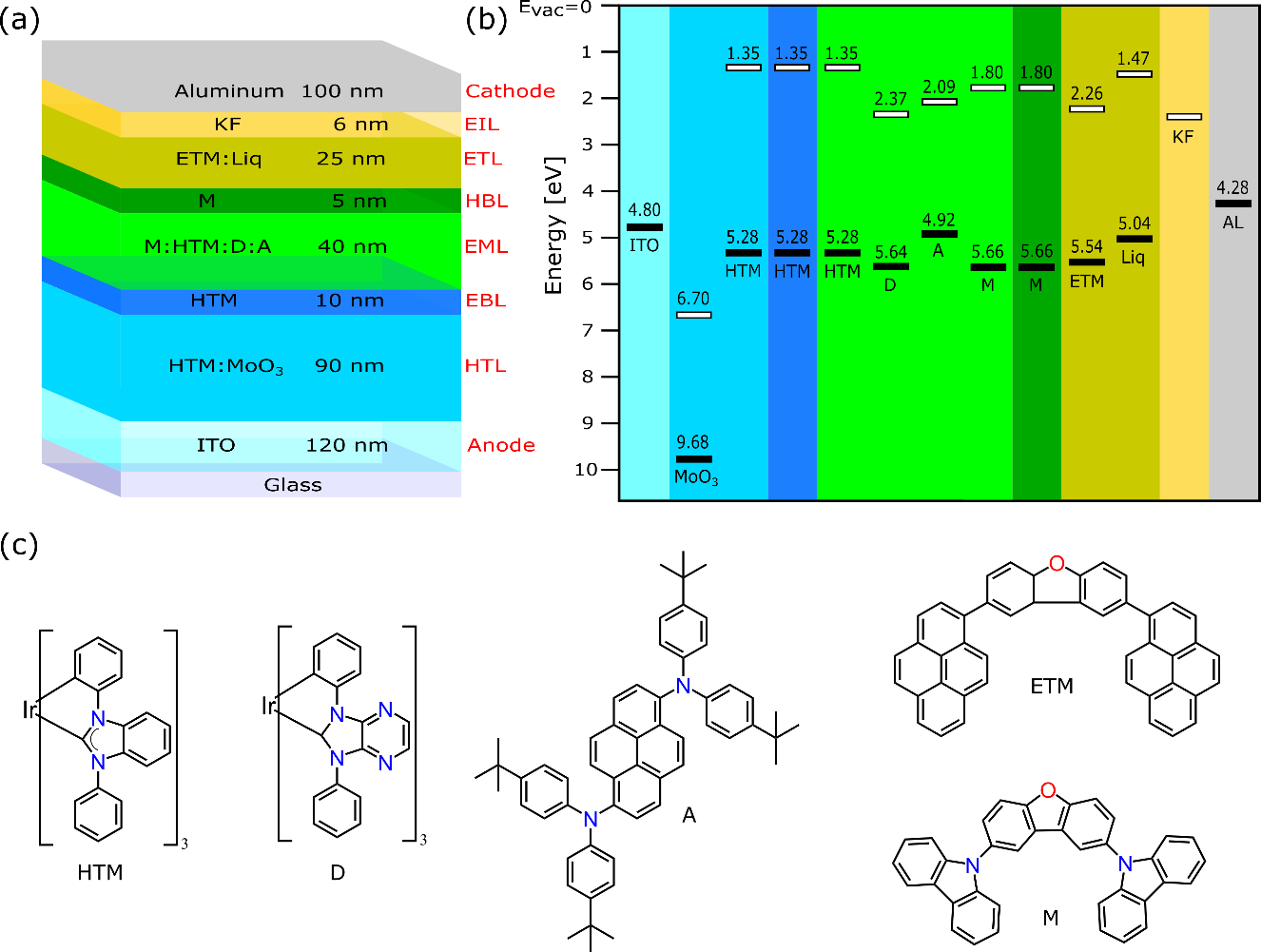


**Supplementary Figure 8**  Organic light emitting diode stack and materials. (a) Device architecture: Cathode, electron injection layer (EIL), electron transport layer (ETL), hole blocking layer (HBL), emission layer (EML), electron blocking layer (EBL), hole transport layer (HTL), Anode. (b) Calculated energy levels of the materials (c) Chemical structures of the materials.

# Comparison of the emission colors

As written in the main text, the emission color of the phosphorescent sensitizer is preserved in the UPSF-OLEDs, as can be seen in the CIE diagram in **Supplementary Figure 9**.


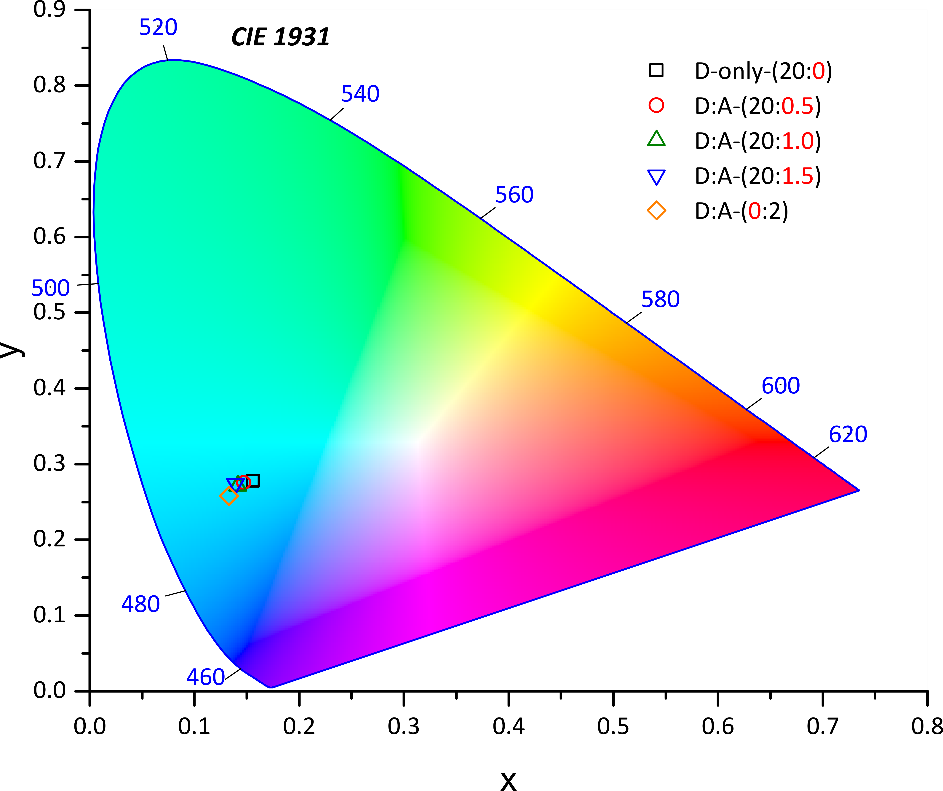


**Supplementary Figure 9** Chromaticity diagram of the unicolored phosphor-sensitized fluorescence diode.

# Blue shift of the donor spectrum

Using **2,5,8,11-Tetra-*tert*-butylperylene (TBPe) as an alternative acceptor**, which possess a different spectral shape of the emission compared to the phosphorescent-only device, the emission color of the UPSF-device is slightly shifted to a deeper blue emission (see **Supplementary Figure 10**). This illustrates that UPSF can also be used to tune the emission color, as mentioned in the main text.


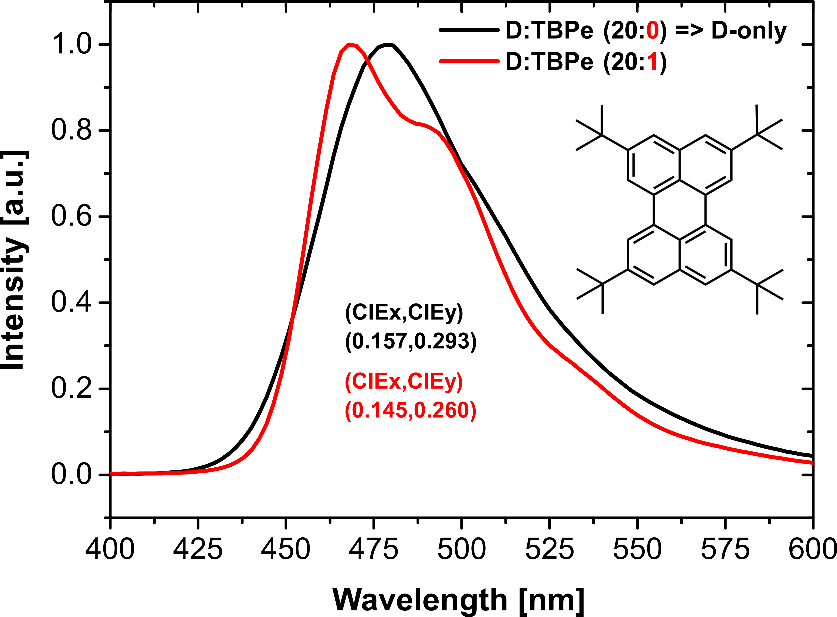


**Supplementary Figure 10** Comparison of electroluminescence spectra. Red curve corresponds to the reference (donor-only) device, while the black curve is for the unicolored phosphor-sensitized fluorescence organic light emitting diode.

# PL and FRET efficiencies

As written in the main text, we concluded that the observed fluorescence of acceptors in the EL- and PL-spectra (see **Figure** **4** (c) (d) of the main text) is due to FRET from the excited donor molecules. To strengthen this conclusion we compared the estimated FRET efficiencies $E_{FRET}$ for the mixed layers to the coefficients of the donor (phosphorescence) and acceptor (fluorescence) from the PL-spectra fits in **Supplementary Figure 11**. The trend of $E_{FRET}$ closely follows the increase in acceptors, reflecting the proportion of fluorescence, with increasing acceptor concentration. This indicates, that the fluorescence is indeed a direct result of FRET from excited donor states.

**
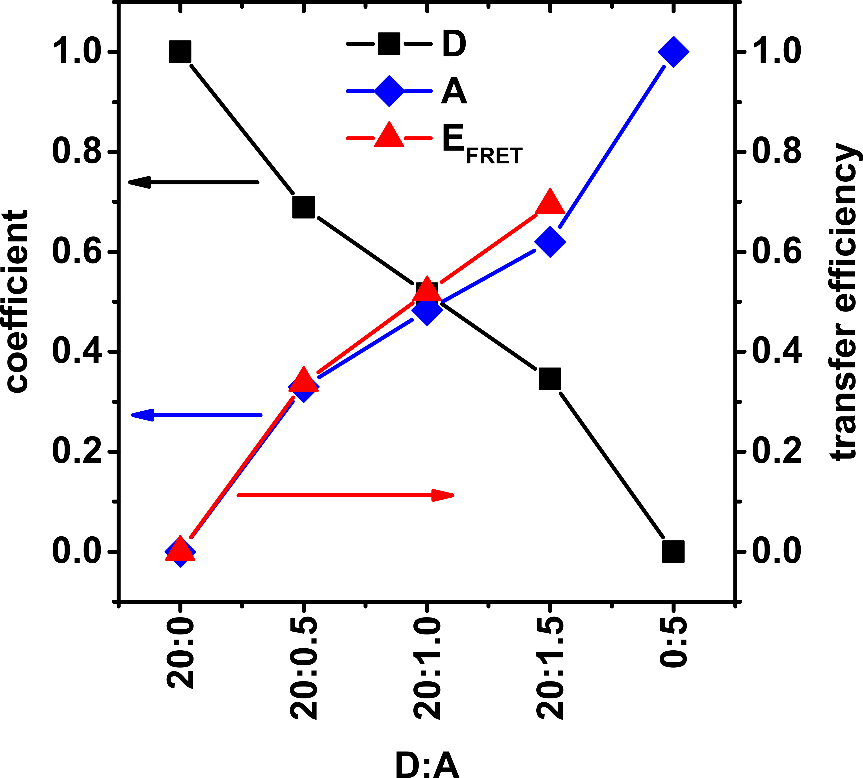
**

**Supplementary Figure 11** Comparison of photoluminescence coefficients and energy transfer (FRET) efficiencies. The donor-only and acceptor-only photoluminescence spectra (**Figure 4**(c, d)) were fitted with three Gaussian peaks. The mixed samples were fitted with linear combinations of the donor-only (D) and acceptor-only (A) fits. The photoluminescence -coefficients (donor and acceptor), reflecting the proportion of phosphorescence and fluorescence, are plotted depending on the A concentration. Additionally, the estimated FRET-efficiencies $E_{FRET}$ are plotted.

# Lifetimes of UPSF OLEDs

Lifetime measurements were performed at the initial current density of 25 mA/cm^2^ (**Supplementary Figure 12** (a)) and initial luminance of 4000 cd/m^2^ (**Supplementary Figure 12** (b)). The LT_70_ values, extracted from the relative decays, are summarized in Table 1 of the main text.


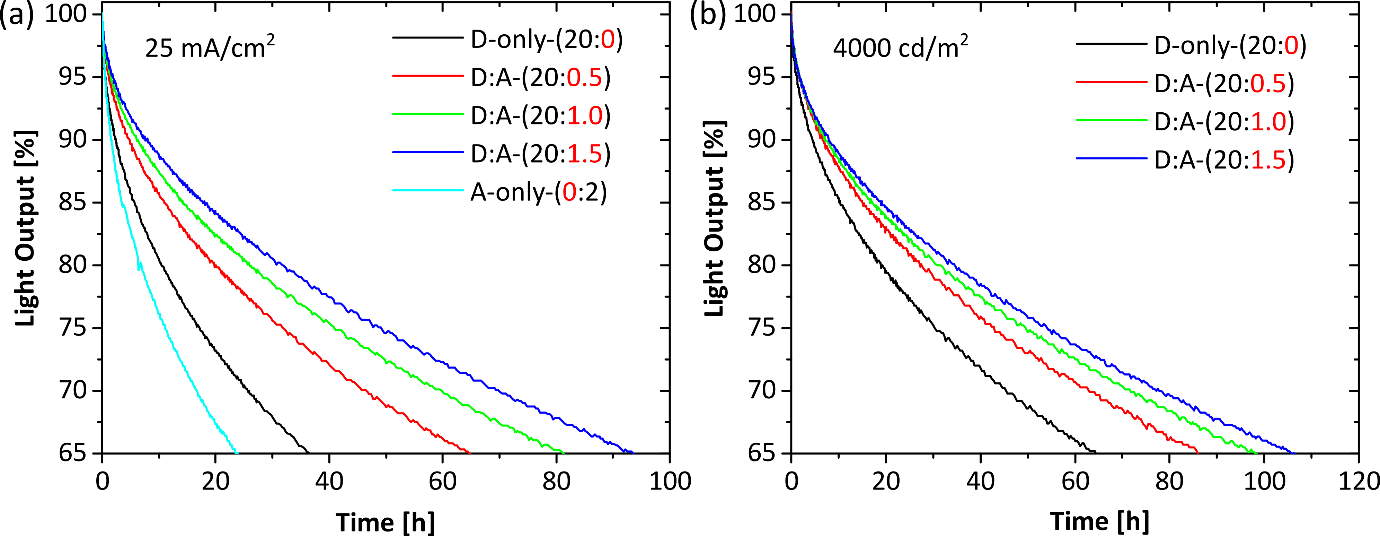


**Supplementary Figure 12** Relative decays of initial luminance (light output) over time. Measurements were performed under constant current at (a) the initial current density of 25 mA/cm^2^ and (b) initial luminance of 4000 cd/m^2^ (b). In (a) an OLED without the donor and 2%-concentration of the acceptor is additionally shown (acceptor-only).

# Lifetime estimation

To compare the lifetime of the 1.5 % OLED measured at an initial luminance of 4000 cd/m^2^ (Table 1 of the main text) to the best (recently published) blue phosphorescent OLEDs,^43,44^ we estimated the LT_80_ value, corresponding to a measurement at 1000 cd/m^2^, by using the relation LT_80_ (1000 cd/m^2^) = LT_80_ (4000 cd/m^2^) x (4000 cd/m^2^ / 1000 cd/m^2^)^n^ with an acceleration factor of n = 1.60.^61,62^

**References**

1. Gómez-Bombarelli, R. *et al.* Design of efficient molecular organic light-emitting diodes by a high-throughput virtual screening and experimental approach. *Nat. Mater.* **15,** 1120–1127 (2016).

2. Hirata, S. *et al.* Highly efficient blue electroluminescence based on thermally activated delayed fluorescence. *Nat. Mater.* **14,** 330–336 (2015).

3. Lee, J. *et al.* Deep blue phosphorescent organic light-emitting diodes with very high brightness and efficiency. *Nat. Mater.* **15,** 92–98 (2015).

4. Baldo, M. A., Adachi, C. & Forrest, S. R. Transient analysis of organic electrophosphorescence. II. Transient analysis of triplet-triplet annihilation. *Phys. Rev. B - Condens. Matter Mater. Phys.* **62,** 10967–10977 (2000).

5. Luo, Y. & Aziz, H. Probing triplet-triplet annihilation zone and determining triplet exciton diffusion length by using delayed electroluminescence. *J. Appl. Phys.* **107,** 94510 (2010).

6. Murawski, C., Leo, K. & Gather, M. C. Efficiency roll-off in organic light-emitting diodes. *Advanced Materials* **25,** 6801–6827 (2013).

7. Zang, F. X. *et al.* Reduced efficiency roll-off in phosphorescent organic light emitting diodes at ultrahigh current densities by suppression of triplet-polaron quenching. *Appl. Phys. Lett.* **93,** 1–4 (2008).

8. van Eersel, H., Bobbert, P. A., Janssen, R. A. J. & Coehoorn, R. Monte Carlo study of efficiency roll-off of phosphorescent organic light-emitting diodes: Evidence for dominant role of triplet-polaron quenching. *Appl. Phys. Lett.* **105,** 143303 (2014).

9. Coehoorn, R., Van Eersel, H., Bobbert, P. A. & Janssen, R. A. J. Kinetic Monte Carlo Study of the Sensitivity of OLED Efficiency and Lifetime to Materials Parameters. *Adv. Funct. Mater.* **25,** 2024–2037 (2015).

10. Uoyama, H., Goushi, K., Shizu, K., Nomura, H. & Adachi, C. Highly efficient organic light-emitting diodes from delayed fluorescence. *Nature* **492,** 234–238 (2012).

11. Tanaka, H., Shizu, K., Miyazaki, H. & Adachi, C. Efficient green thermally activated delayed fluorescence (TADF) from a phenoxazine–triphenyltriazine (PXZ–TRZ) derivative. *Chem. Commun.* **48,** 11392 (2012).

12. Nasu, K. *et al.* A highly luminescent spiro-anthracenone-based organic light-emitting diode exhibiting thermally activated delayed fluorescence. *Chem. Commun.* **49,** 10385 (2013).

13. Endo, A. *et al.* Thermally Activated Delayed Fluorescence from Sn4+-Porphyrin Complexes and Their Application to Organic Light Emitting Diodes - A Novel Mechanism for Electroluminescence. *Adv. Mater.* **21,** 4802–4806 (2009).

14. Nakanotani, H., Masui, K., Nishide, J., Shibata, T. & Adachi, C. Promising operational stability of high-efficiency organic light-emitting diodes based on thermally activated delayed fluorescence. *Sci. Rep.* **3,** 2–6 (2013).

15. Nakanotani, H. *et al.* High-efficiency organic light-emitting diodes with fluorescent emitters. *Nat. Commun.* **5,** 1–7 (2014).

16. Furukawa, T., Nakanotani, H., Inoue, M. & Adachi, C. Dual enhancement of electroluminescence efficiency and operational stability by rapid upconversion of triplet excitons in OLEDs. *Sci. Rep.* **5,** 8429 (2015).

17. Chen, X. K., Zhang, S. F., Fan, J. X. & Ren, A. M. Nature of highly efficient thermally activated delayed fluorescence in organic light-emitting diode emitters: Nonadiabatic effect between excited states. *J. Phys. Chem. C* **119,** 9728–9733 (2015).

18. Lampande, R. *et al.* 45-4: Approach for Attaining Short Exciton Lifetime in Thermally Activated Delayed Fluorescence Emitters. *SID Symp. Dig. Tech. Pap.* **48,** 664–667 (2017).

19. Yao, L., Yang, B. & Ma, Y. Progress in next-generation organic electroluminescent materials: Material design beyond exciton statistics. *Sci. China Chem.* **57,** 335–345 (2014).

20. D’Andrade, B. W. *et al.* High-efficiency yellow double-doped organic light-emitting devices based on phosphor-sensitized fluorescence. *Appl. Phys. Lett.* **79,** 1045–1047 (2001).

21. Baldo, M. A., Thompson, M. E. & Forrest, S. R. High-efficiency fluorescent organic light-emitting devices using a phosphorescent sensitizer. *Nature* **403,** 750–753 (2000).

22. Lei, G., Wang, L. & Qiu, Y. Blue phosphorescent dye as sensitizer and emitter for white organic light-emitting diodes. *Appl. Phys. Lett.* **85,** 5403–5405 (2004).

23. Cheng, G., Zhang, Y., Zhao, Y., Liu, S. & Ma, Y. Improved efficiency for white organic light-emitting devices based on phosphor sensitized fluorescence. *Appl. Phys. Lett.* **88,** 86–89 (2006).

24. Kanno, H., Sun, Y. & Forrest, S. R. White organic light-emitting device based on a compound fluorescent-phosphor-sensitized-fluorescent emission layer. *Appl. Phys. Lett.* **89,** 1–4 (2006).

25. Főrster, T. 10th Spiers Memorial Lecture. Transfer mechanisms of electronic excitation. *Discuss. Faraday Soc.* **27,** 7–17 (1959).

26. Kim, H.-G., Kim, K.-H. & Kim, J.-J. Highly Efficient, Conventional, Fluorescent Organic Light-Emitting Diodes with Extended Lifetime. *Adv. Mater.* **29,** 1702159 (2017).

27. Baek, H.-I. & Lee, C. Simple white organic light emitting diodes with improved color stability and efficiency using phosphorescent and fluorescent emitters. *J. Appl. Phys.* **103,** 124504 (2008).

28. Hou, L. *et al.* Efficient solution-processed phosphor-sensitized single-emitting-layer white organic light-emitting devices: fabrication, characteristics, and transient analysis of energy transfer. *J. Mater. Chem.* **21,** 5312 (2011).

29. Meng, L. C. *et al.* Efficient and color-stable white organic light-emitting diodes based on exciton management and phosphorescent sensitization. *Synth. Met.* **172,** 63–68 (2013).

30. Molt, I. O. *et al.* ( 12 )Metal complexes comprising dazabenzmidazolocarbene ligands and the use thereof in OLEDs, US9487548B2. **2,** (2016).

31. Kaplunov, M. G., Yakushchenko, I. K., Krasnikova, S. S. & Echmaev, S. B. Novel 1,8-bis(diarylamino)pyrenes as OLED materials. *Mendeleev Commun.* **26,** 437–439 (2016).

32. Langer, N. et al. (4). Use of dibenzofurans and dibenzothiophenes substituted by nitrogen-bonded five-membered heterocyclic rings in organic electronics, US9067919B2. (2015).

33. Virgili, T., Lidzey, D. G. & Bradley, D. D. C. Efficient energy transfer from blue to red in tetraphenylporphyrin-doped poly (9, 9-dioctylfluorene) light-emitting diodes. *Adv. Mater.* **12,** 58–62 (2000).

34. Cerullo, G., Stagira, S., Zavelani-rossi, M., Silvestri, S. De & Virgili, T. rster transfer dynamics in tetraphenylporphyrin Ultrafast F o. *Chem. Phys. Lett.* **335,** 27–33 (2001).

35. Nguyen, C., Ingram, G. & Lu, Z.-H. Quantifying Interdopant Exciton Processes in Organic Light Emitting Diodes. *J. Phys. Chem. C* **121,** 3304–3309 (2017).

36. Chang, C. H., Lu, Y. J., Liu, C. C., Yeh, Y. H. & Wu, C. C. Efficient white OLEDs employing phosphorescent sensitization. *IEEE/OSA J. Disp. Technol.* **3,** 193–199 (2007).

37. Ichikawa, M., Aoyama, T., Amagai, J., Koyama, T. & Taniguchi, Y. Dynamic Behavior of Electroluminescence from Phosphor-Sensitized Red Fluorescent Organic Light-Emitting Diodes †. *J. Phys. Chem. C* **113,** 11520–11523 (2009).

38. Xue, Q. *et al.* White organic light-emitting devices with a bipolar transport layer between blue fluorescent and yellow phosphor-sensitized-fluorescent emitting layers. *Synth. Met.* **160,** 829–831 (2010).

39. King, S. M. *et al.* The use of substituted iridium complexes in doped polymer electrophosphorescent devices: The influence of triplet transfer and other factors on enhancing device performance. *Adv. Funct. Mater.* **16,** 1043–1050 (2006).

40. Zhang, D., Song, X., Cai, M. & Duan, L. Blocking Energy-Loss Pathways for Ideal Fluorescent Organic Light-Emitting Diodes with Thermally Activated Delayed Fluorescent Sensitizers. *Adv. Mater.* **1705250,** 1–10 (2017).

41. Kim, K. H., Moon, C. K., Lee, J. H., Kim, S. Y. & Kim, J. J. Highly efficient organic light-emitting diodes with phosphorescent emitters having high quantum yield and horizontal orientation of transition dipole moments. *Adv. Mater.* **26,** 3844–3847 (2014).

42. Jurow, M. J. *et al.* Understanding and predicting the orientation of heteroleptic phosphors in organic light-emitting materials. *Nat. Mater.* **15,** 85–91 (2016).

43. Song, W. & Lee, J. Y. Degradation Mechanism and Lifetime Improvement Strategy for Blue Phosphorescent Organic Light-Emitting Diodes. *Adv. Opt. Mater.* **5,** 1600901 (2017).

44. Lee, J. *et al.* Hot excited state management for long-lived blue phosphorescent organic light-emitting diodes. *Nat. Commun.* **8,** 1–9 (2017).

45. Baumann, T. & Volz, D. 45-3: Invited Paper :Summary for: Recent Progress in Highly Efficient Blue TADF Emitter Materials for OLEDs. *SID Symp. Dig. Tech. Pap.* **48,** 661–663 (2017).

46. Jorgensen, W. L. & Tirado-Rives, J. The OPLS Potential Functions for Proteins. Energy Minimizations for Crystals of Cyclic Peptides and Crambin. *J. Am. Chem. Soc.* **110,** 1657–1666 (1988).

47. Jorgensen, William L., Tirado-Rives, J. *Abstracts of papers*. (American Chemical Society, 1998).

48. Jorgensen, W. L., Maxwell, D. S. & Tirado-Rives, J. Development and testing of the OPLS all-atom force field on conformational energetics and properties of organic liquids. *J. Am. Chem. Soc.* **118,** 11225–11236 (1996).

49. McDonald, N. A. & Jorgensen, W. L. Development of an All-Atom Force Field for Heterocycles. Properties of Liquid Pyrrole, Furan, Diazoles, and Oxazoles. *J. Phys. Chem. B* **102,** 8049–8059 (1998).

50. Poelking, C. *et al.* Characterization of charge-carrier transport in semicrystalline polymers: Electronic couplings, site energies, and charge-carrier dynamics in poly(bithiophene-alt-thienothiophene) [PBTTT]. *J. Phys. Chem. C* **117,** 1633–1640 (2013).

51. Breneman, C. M. & Wiberg, K. B. Determining atom‐centered monopoles from molecular electrostatic potentials. The need for high sampling density in formamide conformational analysis. *J. Comput. Chem.* **11,** 361–373 (1990).

52. Baumann, J. & Fayer, M. D. Excitation transfer in disordered two-dimensional and anisotropic three-dimensional systems: Effects of spatial geometry on time-resolved observables. *J. Chem. Phys.* **85,** 4087–4107 (1986).

53. Kawamura, Y., Brooks, J., Brown, J. J., Sasabe, H. & Adachi, C. Intermolecular interaction and a concentration-Quenching mechanism of phosphorescent Ir(III) complexes in a solid film. *Phys. Rev. Lett.* **96,** 11–14 (2006).

54. Niu, Q. *et al.* Transient electroluminescence on pristine and degraded phosphorescent blue OLEDs. *J. Appl. Phys.* **122,** 185502 (2017).

55. Lee, C.-H., Lee, J.-H., Kim, K.-H. & Kim, J.-J. Unveiling the Role of Dopant Polarity in the Recombination and Performance of Organic Light-Emitting Diodes. *Adv. Funct. Mater.* **1800001,** 1800001 (2018).

56. Bureau, I. WO 2006/128800 A l (74). (2006).

57. Becke, A. D. Density-functional exchange-energy approximation with correct asymptotic behavior. *Phys. Rev. A* **38,** 3098–3100 (1988).

58. Perdew, J. P. Density-functional approximation for the correlation energy of the inhomogeneous electron gas. *Phys. Rev. B* **33,** 8822–8824 (1986).

59. TURBOMOLE V7.0 2015 a development of University of Karlsruhe and Forschungszentrum Karlsruhe GmbH, 1989–2007, TURBOMOLE GmbH since 2007. (2015). Available at: http://www.turbomole.com.

60. Klamt, A. & Schüürmann, G. COSMO: a new approach to dielectric screening in solvents with explicit expressions for the screening energy and its gradient. *J. Chem. Soc., Perkin Trans. 2* 799–805 (1993). doi:10.1039/P29930000799

61. Zhang, Y., Lee, J. & Forrest, S. R. Tenfold increase in the lifetime of blue phosphorescent organic light-emitting diodes. *Nat. Commun.* **5,** 1–7 (2014).

62. Furukawa, K. & Tsujimura, T. New, all-phosphorescent organic light-emitting diodes. *SPIE Newsroom* (2012). doi:10.1117/2.1201209.004469
